# Hierarchical Network with Label Embedding for Contextual Emotion Recognition

**DOI:** 10.34133/2021/3067943

**Published:** 2021-01-06

**Authors:** Jiawen Deng, Fuji Ren

**Affiliations:** Faculty of Engineering, Tokushima University, Tokushima 770-8506, Japan

## Abstract

Emotion recognition has been used widely in various applications such as mental health monitoring and emotional management. Usually, emotion recognition is regarded as a text classification task. Emotion recognition is a more complex problem, and the relations of emotions expressed in a text are nonnegligible. In this paper, a hierarchical model with label embedding is proposed for contextual emotion recognition. Especially, a hierarchical model is utilized to learn the emotional representation of a given sentence based on its contextual information. To give emotion correlation-based recognition, a label embedding matrix is trained by joint learning, which contributes to the final prediction. Comparison experiments are conducted on Chinese emotional corpus RenCECps, and the experimental results indicate that our approach has a satisfying performance in textual emotion recognition task.

## 1. Introduction

As an essential element in human nature, emotions have been wildly studied in psychology. Emotion recognition involves the identification of detailed emotional states, which mainly refer to a wide range of mental states, such as happiness, anger, and fear [1]. Textual emotion recognition (TER) is a kind of fine-grained sentiment analysis. It aims to classify a textual expression into one or several emotion classes depending on the underlying emotion theories employed [2].TER should be the most common application in the field of natural language processing, such as mental health monitoring [3], emotional management [4, 5], sinister tone analysis in social networks [6], and human-computer interaction systems [7]. In recent decades, TER tasks have gained considerable interest in the research community.

Recent researches about TER mainly conducted on sentence level, which are aimed at recognizing subtle emotions based on word and concept-based features extracted from the given sentence. However, emotional expression is complicated, and the same sentence could present different emotions in different contexts. In the absence of contextual information, even humans cannot give confident emotional judgments. Therefore, it is necessary to utilize contextual information for sentence-level emotion recognition.

Given a sentence, its context generally refers to the sentences that appear around it. For example, given a sentence from a blog, its context refers to those sentences that appeared around the current sentence. Given an utterance from a dialogue, its context generally refers to the preceding occurred utterances. Such contextual information has been explored in some preceding works, such as HANs [8], TreeLSTM [9], and CLSTM [10]. Under different circumstances, the contextual sentences of current sentence have different contributions to final prediction, and attention mechanism-based networks are widely utilized to address this problem. Inspired by HANs (Hierarchical Attention Networks), we explore effective encoders for sentence-level encoding and contextual-level encoding to generate more accurate emotion representation expressed in the given sentence.

Emotional expression is very complicated. Some emotions often cooccurred with each other, such as the emotion pair of “Joy” and “Love,” while some are opposed and rarely appear together, such as “Joy” and “Anxiety.” Emotion correlation has always been a significant factor in emotion recognition tasks. To accurately recognize emotions, it is necessary to fully consider the correlations between each emotion.

This paper explores a hierarchical model to learn contextual representations, which encodes the emotional information of a given sentence based on its context. Besides, to realize emotion correlation learning, we trained a label embedding matrix by joint learning, which is beneficial to emotion correlation-based emotion prediction. Therefore, our contributions are summarized below:

1. This paper proposes a hierarchical model to learn contextual representations for sentence-level emotion recognition. We take pretrained language model BERT as the sentence-level encoder and take attention-based bidirectional LSTM as the context-level encoder, which are aimed at learning the emotional information of the given sentence based on its context
2. To give emotion correlation-based prediction, the label embedding matrix is learning by joint learning. Emotion correlation is obtained by calculating the similarity features between sentence representation and each label embedding, which contributes to the final prediction
3. To guarantee the effectiveness of both emotion prediction and label embedding, the proposed network is trained by an assemble training objective. The experimental results indicate that our approach has a satisfying performance in TER task

The rest of this paper is organized as follows: Section 2 presents some related works of textual emotion recognition and contextual modeling.  Section 3 describes the methodology of proposed hierarchical network with label embedding. Experimental results and discussion are shown in Section 4 and Section 5. Finally, Section 6 concludes this paper.

## 2. Related Work

### 2.1. Textual Emotion Recognition

Deep learning-based techniques have achieved significant improvement on TER tasks [11]. Word embedding techniques are aimed at learning latent, low-dimensional representations from the language structure and alleviating the problems of sparse features and high-dimensional representation in traditional bag-of-words models. Some well-established embedding models are wildly used in many NLP tasks, such as Word2vec [12, 13] and GloVe [14]. They are trained on a large scale of unlabeled data and aimed to capture fine-grained syntactic and semantic regularities. Recently, the emergence of pretrained language model opened the pretraining era in the NLP field. The pretrained language models provide useful general knowledge and can be fine-tuned to almost all downstream tasks. CoVe and ELMo [15] generate dynamic and context-sensitive word embedding, by which the same words with different contexts are given different word vectors. They greatly alleviate the occurrence of ambiguity. BERT utilizes a large amount of unlabeled data during the training, which helps the model to learn useful linguistic knowledge. Bert performs well in encoding contextual grammatical knowledge and has achieved satisfactory results in many NLP task.

Emotion label correlation is always a critical problem in TER tasks. Deep canonical correlation analysis (DCCA) performs well in feature-aware label embedding and label-correlation aware prediction [16, 17]. Some studies try to explore the label correlations by transforming multilabel classification tasks into ranking or generation tasks. In [18], they transform multilabel emotion classification tasks into a relevant emotion ranking task, which is aimed at generating a ranked list of relevant emotions according to emotional intensity. In [19], multilabel classification is regarded as a sequence generation task, and Sequence Generation Model (SGM) is proposed to learn label correlations. Some approaches attempt to estimate label correlations by the modification of loss function implicitly. The label-correlation sensitive loss function is first proposed in [20] with the BP-MLL algorithm. Joint binary cross-entropy (JBCE) loss [21] is proposed to train the joint binary neural network (JBNN) to capture label relations. To reduce the computational complexity, partial label dependence can also contribute to this task, which is demonstrated in [22]. A semisupervised multilabel method is proposed in [23], while label correlations are incorporated by modifying the loss function. Multilabel classification and label correlations learning can also be realized in a joint learning framework [24, 25].

### 2.2. Contextual Modeling

Emotion recognition of each sentence in hierarchical texts, such as document or dialogue, highly depends on contextual cues, and context modeling is indispensable. To realize contextual emotion recognition, not only need to model the current sentence but also the contextual sentences, which helps to know the overall emotional tendency of the document or dialogue.

Context modeling architectures can mainly be summarized into two kinds: flatten context modeling and hierarchical context modeling. By flatten context modeling, context sentences and current sentence are concatenated, and all tokens are flattened into a word sequence. This sequence is fed into neural networks for contextual information extraction and final prediction [26, 27]. However, emotions flow naturally in the contextual sentences, and the sequential nature is nonnegligible. Flatten context processing makes the sequence of words too long and ignores the time step. It destroyed the hierarchical structural information of contextual sentences.

The hierarchical structure is a natural characteristic of text: words form sentences and sentences from contexts. This structure knowledge can be incorporated into model architecture for better representation. Inspired by this fact, some works try to stack deep learning architectures to provide specialized understanding at each level [28]. By hierarchical context modeling, each sentence is embedded into a vector representation by sentence-level encoder, and context information is further extracted by hierarchy context encoder [29]. Hierarchical Attention Networks (HANs) is proposed in [8], which mirrors the documents' hierarchical structure. HANs mainly consists of two layers applied at word and sentence level, respectively. In each layer, there is a GRU-based sequence encoder along with an attention layer, which is aimed at paying more attention to important words and sentences that benefit to the final prediction.

## 3. Methodology

### 3.1. Problem Definition

Assume that we have *N* training sample *X* along with their contextual information *C*. Each sample *x* ∈ *X* is often a sentence from the hierarchical text, such as dialogue or blog, and its contextual information *c* = {*c*_1_, *c*_2_, ⋯*c*_*n*_} ∈ *C* often means the preceding *n* sentences appeared before *x*. Each sample *x* is annotated with *K* emotional labels: {*e*_1_, ⋯*e*_*k*_, ⋯*e*_*K*_}, which is denoted as a one hot vector *y* = {*y*_1_, ⋯*y*_*k*_, ⋯*y*_*K*_} ∈ ℝ^1×*K*^, in which *y*_*k*_ = 1 is *x* contains emotion *e*_*k*_ otherwise *y*_*k*_ = 0.

For each sample *x* ∈ *X*, a multilabel emotion recognition model *F* is trained to transform *x* into predicted distributions *p* = {*p*_1_, ⋯*p*_*k*_, ⋯*p*_*K*_} based on its contextual information *c* and then give a final prediction of all possible emotion labels. The function  *F* is denoted as

(1) $$\begin{array}{c} F\left(x,c\right)=\left\{p_{1},\cdots p_{k},\cdots p_{K}\right\}. \end{array}$$

### 3.2. Hierarchical Network with Label Embedding

To model a sentence *x* along with its contextual information *c* = {*c*_1_, *c*_2_, ⋯*c*_*n*_}, the simplest way is to utilizing flatten context modeling, by which *x* and contextual sentence *c* are concatenated as *x*′ = {*c*_1_, *c*_2_, ⋯*c*_*n*_, *x*}, and all tokens in *x*′ are flattened into a word sequence. However, emotions flow naturally in each sentence. Such flatten processing makes the sequence of words too long and ignores the time step, which destroyed the hierarchical structural information. The sequential nature of context is nonnegligible, and such hierarchical information could better contribute to the emotion prediction.

**Figure 1:**
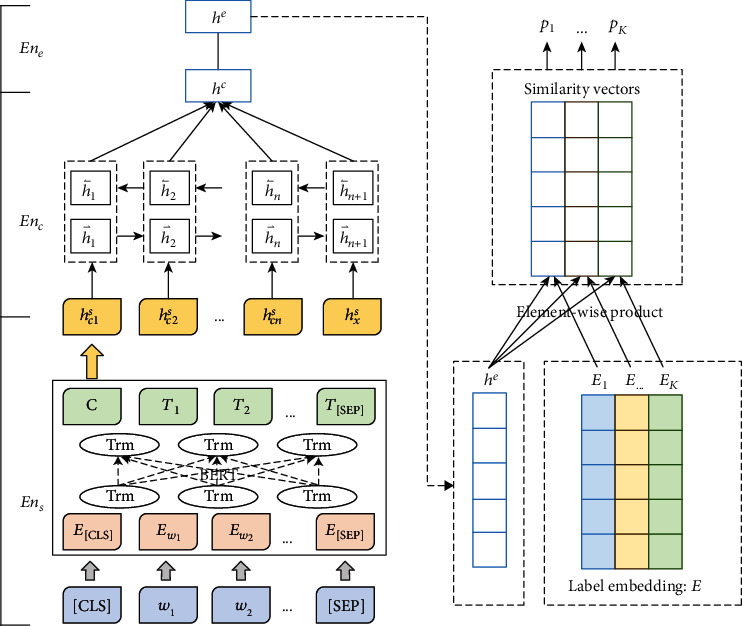
The framework of proposed hierarchical network with label embedding.

Motivated by Hierarchical Attention Networks (HANs), we focus on hierarchical context modeling. Each sentence in *x*′ = {*c*_1_, *c*_2_, ⋯*c*_*n*_, *x*, } is first encoded into sentence-level representation *h*^*s*^ = {*h*_*c*1_^*s*^, *h*_*c*2_^*s*^, ⋯*h*_*cn*_^*s*^, *h*_*x*_^*s*^} by a sentence-level encoder *En*_*s*_, and then, contextual information is further encoded by a hierarchy context encoder. The framework of proposed hierarchical network with label embedding is shown in Figure 1.

#### 3.2.1. Sentence-Level Modeling

At the sentence level, for each sentence *s* = {*w*_1_, *w*_2_, ⋯} in *x*′ = {*c*_1_, *c*_2_, ⋯*c*_*n*_, *x*}, the function *En*_*s*_ encodes *s* into sentence-level representations *h*_*s*_, which is denoted as:

(2) $$\begin{array}{c} h^{s}={En}_{s}\left(s\right). \end{array}$$

Inspired by the pretrained language model and transfer learning techniques, pretrained BERT model [30] is taken as sentence-level encoder *En*_*s*_ in this paper. BERT stands for Bidirectional Encoder Representations from Transformers, and it is designed to pretrain deep bidirectional representations from unlabeled textual data by jointly conditioning on both left and right context in all layers. It remedies the limitation of insufficient training corpora and contributes to syntactic and semantic sentence representation.

In this way, for the sentences in *x*′ = {*c*_1_, *c*_2_, ⋯*c*_*n*_, *x*}, sentence-level representation *h*^*s*^ = {*h*_*c*1_^*s*^, *h*_*c*2_^*s*^, ⋯*h*_*cn*_^*s*^, *h*_*x*_^*s*^} is generated by pretrained BERT model.

#### 3.2.2. Contextual-Level Modeling

At the contextual level, the function *En*_*c*_ encodes the sentence-level representation *h*^*s*^ = {*h*_*c*1_^*s*^, *h*_*c*2_^*s*^, ⋯*h*_*cn*_^*s*^, *h*_*x*_^*s*^} into a context-level representation *h*^*c*^, which is denoted as:

(3) $$\begin{array}{c} h^{c}={En}_{c}\left(h^{s}\right). \end{array}$$

In the proposed model, the function *En*_*c*_ mainly is consisting of two-layer networks: BiGRU (Bidirectional Gated Recurrent Neural Networks) and attention network.

BiGRU is aimed at dealing with the sequential information of contexts. Take sentence-level representation *h*^*s*^ = {*h*_*c*1_^*s*^, *h*_*c*2_^*s*^, ⋯*h*_*cn*_^*s*^, *h*_*x*_^*s*^} as input, the output of the hidden state of BiGRU in each step is *h*_*i*_ = [${\overset{⃑}{h}}_{i}$: ${\overset{⃐}{h}}_{i}$], in which ${\overset{⃑}{h}}_{i}$ and ${\overset{⃐}{h}}_{i}$ are the output of hidden states from forward and backward directions, respectively.

The attention network is aimed at making the network pay more attention to essential contexts. The attention mechanism considers the contributions of previous occurred contextual sentences *c*_*i*_ ∈ *c* to the prediction of current sentence *x*. More attention weight will be assigned to related contexts. Attention weight *a*_*i*_ and weighted emotional feature vector *h*^*c*^ are defined as follows:

(4) $$\begin{array}{c} h^{c}=\underset{i}{\sum }a_{i}h_{i},\\ a_{i}=\frac{\exp\left(e_{i}\right)}{\sum_{k=1}^{n}\exp\left(e_{k}\right)},\\ e_{i}=w_{2}^{T}\left[\sigma \left(w_{1}^{T}∙h_{i}+b_{1}\right)\right]+b_{2}, \end{array}$$

in which *σ* indicates the sigmoid activation function; *w*_1_, *b*_1_, *w*_2_, *b*_2_ indicate the model parameters.

In a typical contextual network, *h*^*c*^ is fed into the classifier for final prediction. The classifier typically consists of a linear transformation. It is followed by a sigmoid operation to normalize the outputs so that each element in will be in the scale of [0,1]. A multilabel neural network is typically trained by minimizing the Binary Cross Entropy (BCE) between the true labels distribution *Y* and predicted distribution *P* as the following:

(5) $$\begin{array}{c} \text{BCE}\left(P,Y\right)=-\frac{1}{N}\overset{N}{\underset{i=1}{\sum }}\overset{K}{\underset{k=1}{\sum }}y_{ik}∙\log\left(p_{ik}\right)+\left(1-y_{ik}\right)∙\log\left(1-p_{ik}\right), \end{array}$$

in which *p*_*ik*_ is the predicted probability of emotion *e*_*k*_ in the *i*th sample, and *y*_*ik*_ is the true label, *y*_*ik*_ ∈ {0, 1}.

Above-mentioned typically network is intuitive and straightforward and wildly utilized in multilabel classification problems. However, emotion recognition is a more complex problem. This typical network with BCE loss function can be less effective and poor generalization due to its ignorance of label correlations. To capture label correlations, a joint learning label embedding network is proposed, which is detailed in Section 3.2.3.

#### 3.2.3. Label Embedding Network

The label embedding is supposed to represent the semantics and relations between emotion labels. The embedding is denoted by

(6) $$\begin{array}{c} E=\left\{E_{1},\cdots E_{k}\cdots E_{K}\right\}\in {\mathbb{R}}^{K\times d}, \end{array}$$

in which *K* is the number of emotion labels, and *d* is the dimension of label embedding. Each row in *E* represents an emotion label.

To make label embedding contribute to the emotion recognition network, the most intuitive way is to compare the emotion representation of contextual input with the label embedding by emotional interaction.

Let the function *En*_*e*_ as emotion projector maps contextual-level representation *h*^*c*^ into emotion representation *h*^*e*^.

(7) $$\begin{array}{c} h^{e}={En}_{e}\left(h^{c}\right)=w_{e}^{T}∙h^{c}+b_{e}. \end{array}$$

Thus, the prediction of all possible emotion labels could be given based on the feature interaction between emotional feature *h*^*e*^ and label embedding matrix *E*. We firstly implement element-wise product operation on *h*^*e*^ and label embedding *E*_*k*_ of each emotion *e*_*k*_:

(8) $$\begin{array}{c} h^{e,k}=h^{e}⊙E_{k},k\in \left[1,K\right]. \end{array}$$

*h* ^*e*,*k*^ denotes the label-aware feature representation, which incorporates the information of input and a particular emotion label *e*_*k*_. In this way, the probability of containing emotion *e*_*k*_ is defined as

(9) $$\begin{array}{c} p_{k}=\sigma \left(w_{c}^{T}∙h^{e,k}+b_{c}\right),\\ \sigma \left(x\right)=\frac{1}{1+e^{-x}}, \end{array}$$

in which *σ* indicates the sigmoid activation function that normalizes the prediction of each emotion *p*_*k*_ in the scale of [0,1]. *w*_c_, *b*_c_ indicate the model parameters. The final prediction is given: *P* = {*p*_1_, ⋯*p*_*k*_, ⋯*p*_*K*_}.

### 3.3. Training Objectives

For multilabel emotion recognition task, the training objective is often based on binary cross-entropy (BCE). However, BCE loss function takes each emotion as an independent individual and does not consider their relationships. Emotion correlation plays an essential role in this task, which makes emotion recognition be a more complex problem than traditional text classification. To guide the model to learn the emotion correlation during the training process, we propose an assembled training objective to consider all aspects.

#### 3.3.1. Training Objective on the Output Layer

To minimize the loss between the true label distribution and the output distribution, label-correlation aware multilabel loss function is applied at the output layer, which is determined as follows:

(10) $$\begin{array}{c} {\text{loss}}_{\text{ML}}=\overset{N}{\underset{i=1}{\sum }}\frac{1}{\left|Y_{i}\right|\left|\overset{¯}{Y_{i}}\right|}\underset{\left(k,l\right)\in Y_{i}\times \overset{¯}{Y_{i}}}{\sum }\exp\left(-\left(p_{k}^{i}-p_{l}^{i}\right)\right), \end{array}$$

where *Y*_*i*_ denotes the set of positive emotions for *i*th sample *x*_*i*_, and $\overset{¯}{Y_{i}}$ denotes the negative emotions. *p*_*k*_^*i*^ and *p*_*l*_^*i*^ are the output possibility of positive emotion *e*_*k*_ and negative emotion *e*_*l*_, respectively. Therefore, training with the above loss function is equivalent to maximizing the difference of (*p*_*k*_^*i*^ − *p*_*l*_^*i*^), which leads the system to output larger values for positive emotions and smaller values for others.

#### 3.3.2. Training Objective on Label Embedding

Given a contextual input *x*, its positive label is *Y*_*i*_ and its negative label is $\overset{¯}{Y_{i}}$, and $Y=Y_{i}\cup \overset{¯}{Y_{i}}$. Emotion representation *h*^*e*^ is learned as in Equation (7). In the proposed network, nonlinear label embedding is utilized in the network to guiding the final prediction *P* by the similarity feature with *h*^*e*^. In this way, we assume that *h*^*e*^ can in turn be used in the training of label embedding by being closer to the embedding of positive emotions while farther to other negative emotions.

To measure the distance of emotion representation *h*^*e*^ and label embedding, cosine embedding loss is utilized.

(11) $$\begin{array}{c} {\text{loss}}_{\text{CosEmbed}}=\overset{N}{\underset{i=1}{\sum }}\frac{1}{K}∙\overset{K}{\underset{k=1}{\sum }}\text{C}\text{osLoss}\left(h_{i}^{e},E_{k}\right), \end{array}$$

(12) $$\begin{array}{c} \text{CosLoss}\left(h_{i}^{e},E_{k}\right)=\left\{\begin{array}{cc} 1-\cos\left(h_{i}^{e},E_{k}\right), & y_{i}\in Y_{\text{pos}},\\ \max\left(0,\cos\left(h_{i}^{e},E_{k}\right)\right), & y_{i}\in Y_{\text{neg}}. \end{array}\right. \end{array}$$

To guarantee label embedding can encode semantic features among labels, we introduce an additional network to recognize each emotion from corresponding label embedding. For each emotion *e*_*i*_, its label embedding is *E*_*i*_. The prediction ${\hat{e}}_{i}$ based on *E*_*i*_ is given as

(13) $$\begin{array}{c} p_{e_{k}}=\text{softmax}\left(W_{ek}∙E_{k}+b_{ek}\right), \end{array}$$

(14) $$\begin{array}{c} {\text{loss}}_{\text{LabelEmbed}}=\frac{1}{K}∙\overset{K}{\underset{k=1}{\sum }}-e_{k}∙\log\left(p_{e_{k}}\right). \end{array}$$

In summary, the assemble training objective of the proposed method is as follows:

(15) $$\begin{array}{c} \text{Loss}\left(x,y\right)={\text{loss}}_{\text{ML}}+{\text{loss}}_{\text{CosEmbed}}+{\text{loss}}_{\text{LabelEmbed}}. \end{array}$$

## 4. Experiments

### 4.1. Dataset

The experiments are conducted on Chinese emotion corpus RenCECps (http://a1-www.is.tokushima-u.ac.jp/member/ren/Ren-CECps1.0/DocumentforRen-CECps1.0.html) to evaluate the proposed architecture. RenCECps is an annotated emotional corpus with Chinese blog texts. The corpus is annotated in document, paragraph, and sentence level [31]. Each level is annotated with eight emotional categories (“Joy,” “Hate,” “Love,” “Sorrow,” “Anxiety,” “Surprise,” “Anger,” and “Expect”).

Our experiments are conducted at sentence level, and the preceding two sentences of the current sentence are taken as the context information. After preprocessing, there are 24310 contextual sentences in training data and 6746 in testing data.

**Figure 2:**
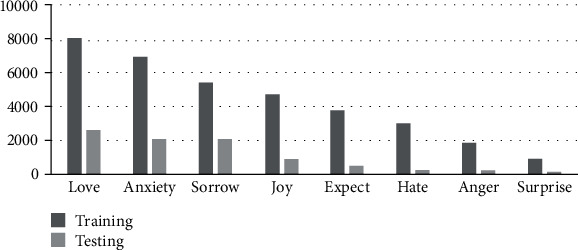
Cumulative number of each emotion in RenCECps.

For each emotion *e*_*i*_, its cumulative number *CN*_*i*_ is calculated.

(16) $$\begin{array}{c} {CN}_{i}=\overset{N}{\underset{\text{n}=1}{\sum }}\left(y_{n,i}=1\right), \end{array}$$

in which *y*_*n*,*i*_ is the annotation of emotion *e*_*i*_ in the *n*th sample, and the statistical results are shown in Figure 2. Label cardinality (LCard) is a standard measure of multilabeledness and means the average number of emotions concluded per sentence of the corpus and [32]. In RenCECps, LCard is 1.4468.

### 4.2. Evaluation Metrics

The global performance is evaluated by micro- and macro-F1 score. F1 score is the harmonic mean of precision and recall. Micro-F1 score gives each sample the same importance, while macro-F1 score takes all classes as equally important.

Some popular evaluation measures typically utilized in multilabel classification are utilized to measure the efficiency of proposed methods. Hamming Loss (HL) is the fraction of labels that are incorrectly predicted. Coverage evaluates how far it is needed to go down the ranked emotion list to cover all the relevant emotions in the instance. One Error (OE) evaluates the fraction of sentences whose top-ranked emotion is not in the relevant emotion set. Ranking Loss (RL) evaluates the average fraction of label pairs that are reversely ordered for instance.

### 4.3. Experimental Details

For a given sentence, its preceding two sentences are taken as contextual sentences. There are total 8 emotion labels annotated for each sentence, and the dimension of label embedding is set to 256. The dimension of hidden state of GRU cell is set to 768/2, and 768 is the dimension of sentence-level embedding.

During the model training, the learning rate is set to 2*e*-5, and the batch size is set to 128. Adam optimization method is applied to train the model by minimizing the proposed training objective.

### 4.4. Baselines

In this section, we report the experimental results of our proposed method and baseline models. Additionally, we analyze the influence of training objectives on output layer and label embedding.

We compare our proposed model with six baseline methods as follows.

*(1) RERc* [18]: a novel framework based on relevant emotion ranking to identify multiple emotions and produce the rankings of relevant emotions from text.

*(2) HANs* [8]: it has a hierarchical structure that mirrors the hierarchical structure of documents and has two levels of attention mechanisms applied at the word-and sentence-level. In our experiments, sentence-level encoder of HANs is replaced by pretrained BERT model.

*(3) EDL* [33]: Emotion Distribution Learning, it learns a mapping function from texts to their emotion distributions describing multiple emotions and their respective intensities based on label distribution learning.

*(4) EmoDetect* [34]: it outputs the emotion distribution based on a dimensionality reduction method using nonnegative matrix factorization, which combines several constraints such as emotions bindings, topic correlations, and emotion lexicons in a constraint optimization framework.

*(5) ML-KNN* [35]: Multi-Label k-Nearest Neighbor, which adapts the traditional *k*-nearest neighbor (KNN) algorithm to deal with multilabel data.

*(6) Rank-SVM* [36]: adapts maximum margin strategy to deal with multilabel data and focuses on distinguishing relevant from irrelevant while neglecting the rankings of relevant ones.

The comparison experiments of baseline HANs are implemented based on the open-source codes shared on GitHub, and the results of other baselines are adopted from the published papers.

## 5. Experimental Results and Discussions

### 5.1. Results Analysis

The experimental results of our model compared with the baselines on the RenCECps dataset are shown in Table 1. The best result on each metric is in italics.

As the results shown in Table 1, it indicates that our proposed method significantly outperforms baseline models to a great extent and achieves satisfactory performance on RenCECps dataset. For example, compared to the baseline RERc, our model achieves an improvement of 10.73% micro-F1 score. On multilabel evaluation measures, our model achieves a reduction of 46.15% ranking loss and 21.78% one error. Compared to other baselines, our model achieved satisfactory results as well, which demonstrated the effectiveness of the proposed method.

### 5.2. The Effectiveness of Label Embedding Layer

Our proposed model is an extension of the baseline of HANs. In our experiments, sentence-level encoder of HANs is replaced by pretrained BERT model. Therefore, by comparing the results of these two models, it can be revealed whether the addition of label embedding layer is effective on the subtask of emotion correlation learning.

As we can see from the results shown in Table 1, the proposed model significantly outperforms baseline HANs, which achieves the improvement of micro-F1 score from 0.5573 to 0.5665 and macro-F1 score from 0.4003 to 0.4186. On multilabel evaluation measures, our model achieves a reduction of ranking loss from 0.1136 to 0.1131, one error from 0.3623 to 0.3559, and hamming loss from 0.2075 to 0.1998.

**Figure 3:**
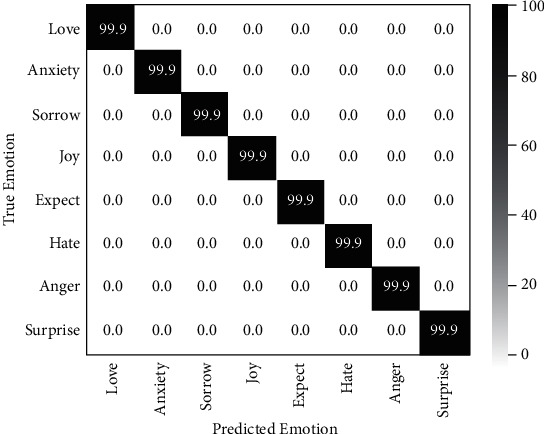
The prediction probability given by label embedding matrix.

Both the proposed method and baseline HANs give prediction based on the contextual representation learned from a hierarchical network. HANs directly fed it into output layer for final prediction, which mainly consists of a fully connected layer and an activate function like sigmoid. This implementation is intuitive and straightforward, and it is also a common processing method in most multilabel classification tasks. However, such implementation treats emotion recognition task as a general text classification task. It does not consider the correlation between emotion labels, such as the probability of cooccurrence of “Love” and “Happy” is higher than that of “Love” and “Sad.” In our proposed model, label embedding space is introduced for emotion correlations learning. The final prediction is based on the interaction of the emotion representation of input text and the label embedding matrix. To guarantee that the label embedding matrix learned the semantic features among labels, training objective on label embedding is utilized to guide the training. The predicted probability given by label embedding matrix, as Equation (13), is visualized in Figure 3. The results in the figure clearly show that the label embedding matrix can accurately predict the corresponding emotion, which means that the emotional information of each label has been actually learned in the label embedding matrix.

### 5.3. The Effectiveness of Training Objectives

As described in Section 3.3, we proposed an assembled training objective to realize the joint learning of both emotion recognition task and label embedding task. To evaluate the effectiveness of training objectives and label embedding network, we train the proposed model with different training objectives. The results are shown in Table 2. The symbols “M,” “C,” and “L” denote the loss function of multilabel loss, as in Equation (10), cosine embedding loss, as in Equation (11), and label embedding loss, as in Equation (14), which are utilized for training.

From Table 2, compared with the assembled training objective (“M+C+L”), the proposed model with only multilabel loss (“M”) on output layer achieves a reduction of 2.22% micro-F1 and 1.39% macro-F1 and an improvement of 12.37% ranking loss, 6.41% one-error, and 4.52% coverage. It suggests that the proposed ensemble training objective can contribute to the classification improvement.

The experimental results of the proposed model trained on “M+C” and “M” indicate the contribution of cosine embedding loss on the training of the label embedding matrix. Cosine embedding loss guides the training of label embedding by making the emotion representation of input being closer to the embedding of positive emotion labels while farther to other negative emotion labels.

The comparison results of the proposed model trained on “M+L” and “M” indicate that the addition of label embedding loss is effective. Label embedding loss guarantees that the trained label embedding matrix is able to encode semantic features among emotion labels.

## 6. Conclusions

In this paper, we proposed a hierarchical network with label embedding for contextual emotion recognition. Our method involves hierarchically encoding the given sentence based on its contextual information and training a label embedding matrix with an assembled training objective to realize emotion correlation learning. The experimental results show the strong ability of the proposed method to learn emotional features for contextual emotion recognition. In the future, it shall be interesting to incorporate background resources, such as emotion lexicon and knowledge graph, to make the system more satisfactory and robust.

## Figures and Tables

**Table 1:** Experimental results in RenCECps.

Metrics	Ours	RERc	HANs	EDL	EmoDetect	ML-KNN	Rank-SVM
Micro-F1 (↑)	*0.5665*	0.5116	0.5573	0.4620	0.4552	0.4720	0.4962
Macro-F1(↑)	*0.4186*	0.4161	0.4003	0.3923	0.3622	0.3632	0.3965
Ranking loss (↓)	*0.1132*	0.2102	0.1136	0.2589	0.2781	0.2928	0.3024
One-error (↓)	*0.3559*	0.4550	0.3623	0.5227	0.5352	0.5543	0.5606
Coverage (↓)	2.1272	*2.1268*	2.1272	2.1699	2.8956	2.4448	2.5962
Hamming loss (↓)	*0.1998*	0.2014	0.2075	0.2102	0.2202	0.2409	0.2585

**Table 2:** Ablation experimental results with different training objectives.

Metrics	M+C+L	M+C	M+L	M
Micro-F1 (↑)	*0.5665*	0.5655	0.5570	0.5539
Macro-F1(↑)	0.4186	*0.4246*	0.4156	0.4128
Ranking loss (↓)	*0.1132*	0.1209	0.1194	0.1272
One-error (↓)	*0.3559*	0.3734	0.3719	0.3787
Coverage (↓)	*2.1272*	2.1778	2.1638	2.2234
Hamming loss (↓)	0.1998	0.1959	0.2040	*0.1957*

“M”: multilabel loss; “C”: cosine embedding loss; “L”: label embedding loss.
